# Encapsulation in Calcium Alginate Beads Regulates Growth, Release and Viability of Probiotic Bacteria Through Protective Microenvironments

**DOI:** 10.3390/gels12060518

**Published:** 2026-06-10

**Authors:** Weichen Gong, Luciano Arellano-Arriagada, Julio Villena, Keita Nishiyama, Haruki Kitazawa

**Affiliations:** 1 Laboratory of Animal Food Function, Graduate School of Agricultural Science, Tohoku University, Sendai 980-8577, Japankeita.nishiyama.a6@tohoku.ac.jp (K.N.); 2 Livestock Immunology Unit, International Education and Research Center for Food and Agricultural Immunology (CFAI), Graduate School of Agricultural Science, Tohoku University, Sendai 980-8577, Japan; 3 Laboratory of Respiratory Immunology (LaRI), Division of Animal Immunology and Omics, International Education and Research Center for Food and Agricultural Immunology (CFAI), Graduate School of Agricultural Science, Tohoku University, Sendai 980-8577, Japan

**Keywords:** encapsulation platform, controlled release, biomaterial-assisted viability maintenance

## Abstract

**Aims**: Our aim was to evaluate calcium alginate beads as a physical cultivation platform for probiotic bacteria and investigate their effects on bacterial growth localization, release dynamics, and viability under different environmental conditions. **Methods and Results**: Calcium alginate beads were fabricated using sodium alginate at different concentrations (1.0–2.0%, *w*/*v*), and their structural stability, permeability, and pH resistance were assessed. *Lacticaseibacillus paracasei* was used as the primary model organism. Among the tested formulations, 2.0% alginate exhibited the lowest permeability (~40%) compared with 1.0% and 1.5% alginate (~60%) and maintained structural integrity under alkaline conditions (pH 11–12). Encapsulated *L. paracasei* proliferated predominantly within the bead matrix, while less than 1% of total bacteria were released into the surrounding medium during the first 12 h and approximately 8% released after 24 h. Encapsulation also maintained higher bacterial viability over 24–48 h compared with conventional liquid culture conditions. Encapsulated cells could be efficiently recovered using sodium citrate without marked loss of viability. In addition, encapsulation enabled the growth of *Bifidobacterium animalis* subsp. *lactis* under aerobic incubation conditions, suggesting the formation of locally protective microenvironments within the gel matrix. **Conclusions**: Calcium alginate beads function as controllable microenvironmental regulators that influence bacterial growth, release behavior, and long-term viability. These findings suggest that alginate-based encapsulation systems may provide a simple and scalable platform for controlled microbial cultivation and probiotic-related applications.

## 1. Introduction

The application of lactic acid bacteria in fermented foods and functional products has expanded rapidly in recent years [1]. However, maintaining bacterial viability and stability during cultivation and processing remains a persistent challenge. In conventional liquid culture systems, bacterial populations typically undergo rapid expansion followed by a decline in viability due to nutrient depletion, acid accumulation, and environmental stress, leading to variability in performance and reduced stability of final products [2].

*Lacticaseibacillus paracasei* is widely used in fermentation processes and has been investigated in probiotic-related contexts [3,4]. However, similar to other lactic acid bacteria, its viability is sensitive to environmental conditions, particularly during extended cultivation [5,6]. Current strategies to improve performance have mainly focused on strain selection, medium optimization, and co-culture systems, while comparatively less attention has been given to the role of the physical cultivation environment. Encapsulation technologies have therefore attracted considerable interest as strategies to improve probiotic stability and functionality. Recent reviews have highlighted alginate-based hydrogels as one of the most widely used food-grade encapsulation systems due to their biocompatibility, mild gelation conditions, and ability to protect microorganisms from environmental stress during processing, storage, and gastrointestinal transit [7].

Alginate-based hydrogels have been extensively studied as encapsulation materials due to their biocompatibility, mild gelation conditions, and food-grade safety [8]. Alginate-based encapsulation has been extensively used to improve probiotic stability during gastrointestinal transit, storage, freeze-drying, and incorporation into food products [9]. Many studies have further improved alginate beads by adding prebiotics, gelatin, chitosan, starch, or multilayer coatings to enhance acid resistance, bile tolerance, and controlled release [10,11,12]. Recent advances have further expanded the application of alginate hydrogels toward multifunctional probiotic delivery systems, including stimuli-responsive materials, multilayer encapsulation platforms, and engineered hydrogel microenvironments. These systems have primarily been developed to improve probiotic survival and delivery efficiency [13,14].

Previous studies have primarily focused on their protective role during gastrointestinal delivery or storage [12]. Alginate hydrogels also provide a three-dimensional microenvironment that can spatially confine bacterial cells while allowing diffusion of nutrients and metabolites [15]. This raises the possibility that such systems could influence bacterial growth dynamics and release behavior during cultivation. Comparatively fewer studies have quantified how calcium alginate beads affect bacterial growth localization, time-dependent release, recoverability, and viability during active cultivation. Although several studies have investigated probiotic growth within hydrogel matrices, including in situ cultivation approaches designed to increase bacterial loading capacity, quantitative analyses of bacterial localization, release dynamics, recoverability, and viability during active cultivation remain limited. For example, Huang et al. demonstrated that probiotics can proliferate within alginate hydrogel beads and achieve high cell densities during in situ cultivation; however, bacterial confinement behavior and release kinetics were not systematically evaluated [16,17,18].

In this study, we evaluated calcium alginate beads as a physical cultivation environment for *L. paracasei*. By optimizing alginate concentration, we assessed bead stability, permeability, and their impact on bacterial growth, release dynamics, and viability. Our results suggest that alginate beads can modulate bacterial behavior during cultivation and may serve as a controllable platform for studying and potentially regulating microbial growth in confined environments. Therefore, the aim of this study was to evaluate calcium alginate beads not only as probiotic encapsulation carriers but also as controllable cultivation microenvironments. Using *L. paracasei* as a model organism, we investigated how alginate concentration influences bead stability, permeability, bacterial growth localization, release kinetics, recoverability, and long-term viability. In addition, *Bifidobacterium animalis* subsp. *lactis* was used to explore whether encapsulation could support bacterial growth under aerobic conditions. Through these analyses, we sought to clarify how alginate hydrogels regulate microbial behavior during active cultivation rather than solely during storage or gastrointestinal delivery.

## 2. Results and Discussion

### 2.1. Optimization of Sodium Alginate Concentration for Stable Bead Formation

**Figure 1:**
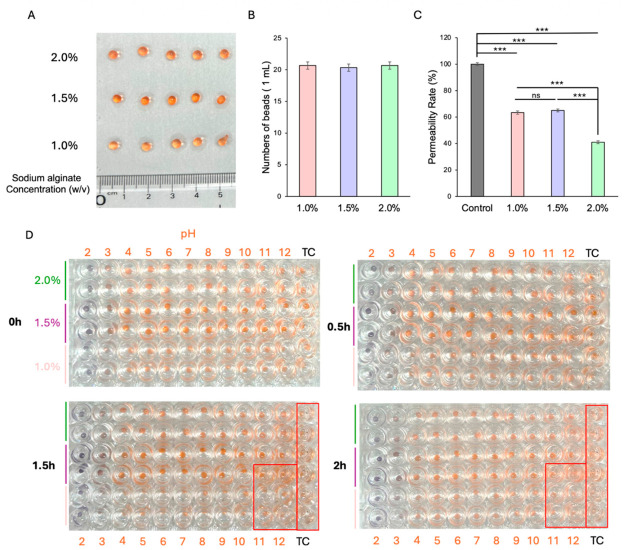
Fabrication and physicochemical characterization of calcium alginate beads: (**A**) Representative images of calcium alginate beads prepared from 1.0%, 1.5%, and 2.0% (*w*/*v*) sodium alginate solutions. (**B**) Quantification of bead yield produced from 1 mL alginate solution. (**C**) Relative permeability of alginate hydrogels with different concentrations, measured using a hydrogel-coated filter system. Data are presented as mean ± SD (n = 3 biological replicates). Statistical significance was determined by one-way ANOVA followed by Tukey’s multiple comparison test. ns, not significant; *** *p* < 0.001. (**D**) pH stability of calcium alginate beads after 1.5 h incubation in solutions with varying pH values. Beads prepared with 2.0% alginate retained structural integrity under both acidic and alkaline conditions, whereas lower concentrations exhibited partial dissolution at high pH. TC, trisodium citrate.

Sodium alginate solutions exhibit high viscosity, which makes complete dissolution difficult without thermal sterilization. However, high-temperature sterilization may lead to random cleavage of alginate polymer chains, resulting in heterogeneous gel formation [19]. Calcium alginate beads were generated using sodium alginate solutions at concentrations of 1.0%, 1.5%, and 2.0% (*w*/*v*). Among these conditions, 2.0% alginate consistently produced beads with more uniform size and well-defined spherical morphology, whereas beads prepared from lower concentrations exhibited deformation and reduced structural integrity (Figure 1A).

To estimate bead volume, the number of beads generated per milliliter of alginate solution was quantified. Approximately 20 beads were formed per mL, corresponding to an estimated volume of ~50 μL per bead (Figure 1B). Permeability analysis using crystal violet diffusion indicated that 1.0% and 1.5% alginate hydrogels exhibited higher permeability (~60%), whereas permeability decreased to approximately 40% in 2.0% alginate, suggesting the formation of a denser gel network at higher polymer concentrations (Figure 1C).

These results indicate that increasing alginate concentration improves structural stability. These findings are consistent with previous reports showing that higher alginate concentrations produce hydrogels with greater mechanical strength and stiffness. Therefore, 2.0% alginate was selected for subsequent experiments.

Previous studies have demonstrated that increasing alginate content enhances bead rigidity and mechanical resistance while decreasing permeability to solutes and oxygen [20]. Similar concentration-dependent effects on hydrogel stability and diffusion behavior have also been reported in probiotic encapsulation systems and immobilized-cell fermentation studies [21]. However, most previous studies primarily evaluated these properties in the context of gastrointestinal protection or storage stability [11,12,22], whereas the present study further examined how these physicochemical differences influence bacterial localization, release dynamics, and long-term cultivation behavior.

### 2.2. Stability of Calcium Alginate Beads Under Different pH Conditions

The structural stability of alginate beads was evaluated under acidic and alkaline conditions. Beads prepared from 1.0% and 1.5% alginate showed partial dissolution under strongly alkaline conditions (pH = 11–12), whereas 2.0% alginate beads maintained their spherical structure under the same conditions (Figure 1D).

These observations suggest that higher alginate concentrations enhance resistance to extreme pH environments, which may be advantageous for applications involving variable environmental conditions.

### 2.3. Growth Behavior of L. paracasei Within Alginate Beads

**Figure 2:**
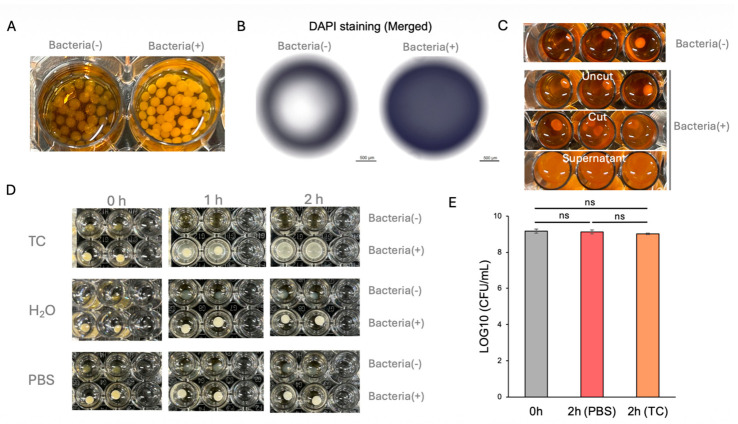
Cultivation of *L. paracasei* within alginate beads and citrate-mediated bacterial release. (**A**) Visual appearance of *L. paracasei*-containing alginate beads after cultivation in MRS medium. (**B**) DAPI staining of *L. paracasei*-containing and sterile alginate beads. (**C**) Growth assessment of bacteria released from intact or mechanically cut beads after 20 h incubation. (**D**) Complete dissolution of calcium alginate beads following treatment with sodium citrate for 2 h. TC, trisodium citrate. (**E**) Viability assessment of bacteria recovered after citrate-mediated dissolution. Data are presented as mean ± SD (n = 3 biological replicates). Statistical significance was determined by one-way ANOVA followed by Tukey’s multiple comparison test. ns, not significant.

*L. paracasei* JCM 2769 was selected as a model organism for encapsulation studies due to its ready availability and robust growth characteristics, making it suitable for routine laboratory cultivation and experimental reproducibility [23]. When *L. paracasei* was encapsulated in alginate beads and cultured in MRS medium, the beads gradually became turbid, while the surrounding medium remained relatively clear (Figure 2A,B). This observation is consistent with bacterial proliferation occurring largely within the beads, although this interpretation is based on indirect visual assessment. Direct microscopic measurements showed that the beads had an initial diameter of approximately 4.2 mm, which increased significantly to approximately 4.9 mm after 12 h of incubation in MRS medium (Figure S1). Viable bacteria were detected in both the beads and the surrounding medium, indicating that cells were able to grow within the beads and partially migrate into the external environment over time (Figure 2C).

Sodium citrate was used as a chelating agent to sequester Ca^2+^ ions from the calcium alginate matrix, thereby disrupting the crosslinked gel structure. Thus, encapsulated bacteria could be recovered by dissolving calcium alginate beads using sodium citrate. Treatment with sodium citrate for 2 h resulted in complete dissolution of the beads and efficient release of encapsulated bacteria (Figure 2D). Following dissolution, viable bacteria were successfully recovered without a marked decrease in CFU counts (Figure 2E). These results indicate that citrate-mediated dissolution enables efficient recovery of viable cells from the beads under the tested conditions.

The ability to recover viable bacteria through citrate-mediated dissolution indicates that the encapsulation process is compatible with downstream applications. While short-term viability was preserved, additional studies would be needed to evaluate potential effects on physiological functions or long-term stability. Numerous previous studies have shown that alginate encapsulation improves probiotic survival during storage, freeze-drying, or simulated gastrointestinal exposure. Our findings are generally consistent with these observations, as encapsulated bacteria maintained substantially higher viability than free-living cultures after prolonged cultivation [24].

Regarding the sterilization of alginate solutions, ultraviolet irradiation (UV) was used in this study to avoid thermal degradation of the polymer [25]. No bacterial colonies were detected when blank UV-treated alginate beads were incubated, dissolved, and plated under the same experimental conditions, providing additional evidence that the sterilization procedure was effective for the present study. However, it should be noted that UV sterilization may not achieve complete sterilization in all contexts. Filtration-based sterilization was not applied in this study due to the high viscosity of 2% alginate solutions, which makes passage through standard 0.2 µm filters technically challenging. Instead, sterility was evaluated experimentally, and no bacterial growth was observed in control cultures using UV-treated alginate solutions. Alternative sterilization approaches may be explored in future studies.

### 2.4. Release Dynamics of Encapsulated Bacteria

**Figure 3:**
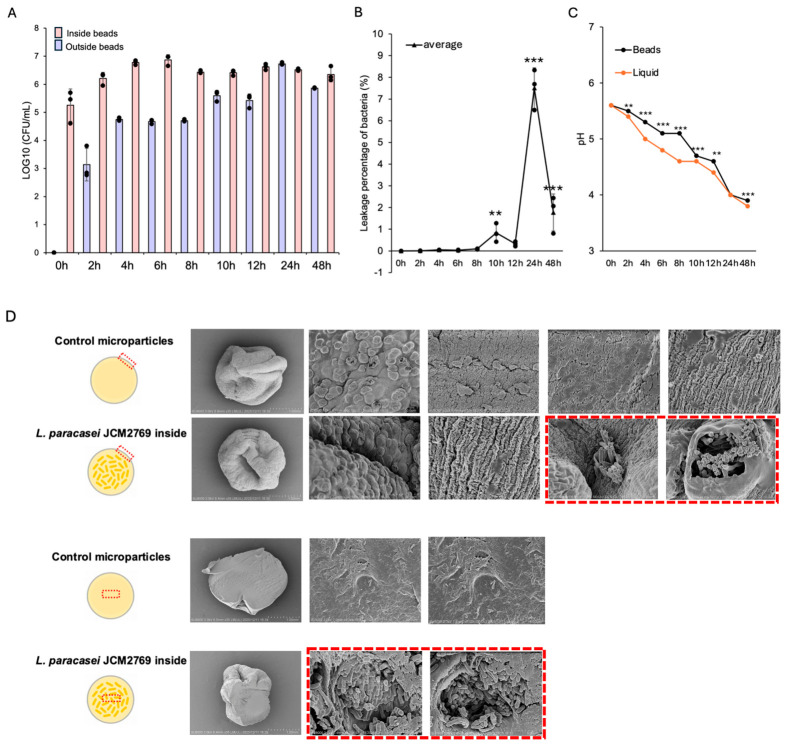
Release kinetics and structure of *L. paracasei* within alginate beads: (**A**) Quantification of bacterial distribution inside and outside alginate beads over time. (**B**) Quantitative analysis of bacterial release kinetics over time. Statistical significance was determined by comparing each time point with the 0 h control. (**C**) Changes in culture medium pH during cultivation of encapsulated bacteria compared with conventional liquid culture. Data (**B**,**C**) are presented as mean ± SD (n = 3 biological replicates). Statistical significance was determined by one-way ANOVA followed by Tukey’s multiple comparison test. ** *p* < 0.01, *** *p* < 0.001. (**D**) Scanning electron microscopy images showing bacterial penetration of the bead surface and bacterial growth within the internal pore structure of alginate beads after 24 h cultivation.

The detection of viable bacteria in the supernatant prompted us to quantify the release kinetics of *L. paracasei* from alginate beads. One bead was incubated in 1 mL of MRS medium, and at defined time points, both the culture supernatant (50 μL) and the bead itself (dissolved with sodium citrate) were collected for CFU enumeration (Figure 3A). Quantitative analysis revealed an exceptionally slow release profile. During the first 12 h of incubation, less than 1% of total bacteria were detected in the surrounding medium. After 24 h, approximately 8% of the bacterial population had diffused out of the beads (Figure 3B). Each time point was analyzed using an independent culture sample, thereby avoiding potential artifacts associated with repeated sampling or progressive depletion of culture volume.

Measurements of culture pH demonstrated that acidification proceeded at a rate comparable to that observed in conventional liquid cultures (Figure 3C), indicating that metabolic products freely diffused through the alginate matrix. Scanning electron microscopy (SEM) further confirmed these findings. Bacteria were observed both within the internal pores of the beads and at sites penetrating the bead surface, providing direct structural evidence for localized bacterial migration through the alginate network (Figure 3D).

Overall, these results indicate that bacterial release from alginate beads was limited during early cultivation and increased progressively, suggesting a time-dependent release behavior.

Encapsulation of *L. paracasei* was associated with spatial confinement of bacterial growth within the beads. Similar localized microcolony formation has previously been reported in hydrogel-based immobilization systems, including pectin and alginate matrices designed for probiotic delivery [26]. Heumann et al. demonstrated that hydrogel microbeads can support dense bacterial microcolonies while physically restricting large-scale bacterial dispersion [27]. Our observations are generally consistent with these reports. However, in contrast to previous studies that mainly focused on intestinal delivery or colonization efficiency, the present study quantitatively monitored bacterial distribution inside and outside the beads during active cultivation. The combination of turbidity observation, CFU quantification, and SEM analysis suggests that calcium alginate beads function not merely as passive carriers but as spatially regulating cultivation environments. An interesting observation in the present study was that bacterial proliferation occurred predominantly within the alginate bead matrix rather than in the surrounding medium. This finding suggests that the hydrogel structure provided a confined cultivation environment that retained bacterial cells while still allowing sufficient nutrient exchange to support growth. Although nutrient gradients, oxygen gradients, and metabolite distributions were not directly measured, the observed localization pattern indicates that alginate beads may influence the spatial organization of bacterial populations during cultivation. Future studies incorporating nutrient transport analysis, microsensor measurements, and imaging-based metabolic profiling will be required to further characterize the microenvironmental conditions within the beads.

### 2.5. Viability of L. paracasei in Beads Compared with Liquid Culture

To compare bacterial survival under encapsulated and free-living conditions, CFU counts were determined for liquid cultures and alginate beads after 24 and 48 h of incubation. For quantitative consistency, 50 μL of liquid culture was compared with one bead (approximately 50 μL alginate volume).

**Figure 4:**
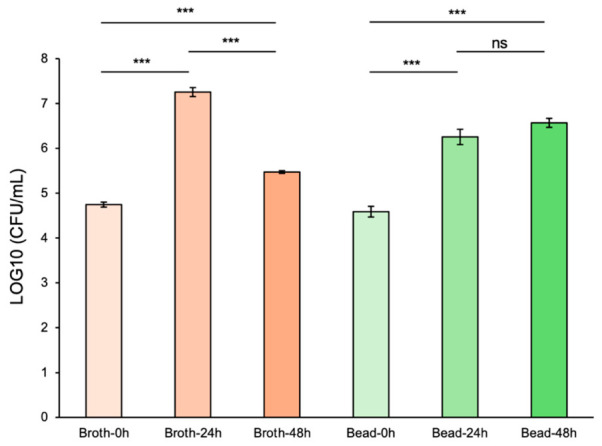
Enhanced long-term viability of *L. paracasei* cultured within alginate beads. Comparison of bacterial viability between conventional liquid culture and alginate bead encapsulation after 24 and 48 h of cultivation. The experiment was independently repeated three times (biological replicates). Data are presented as mean ± SD (n = 3 biological replicates). Statistical analysis was performed using one-way analysis of variance (ANOVA), followed by Tukey’s multiple comparisons test. ns, not significant; *** *p* < 0.001.

Although free-living cultures exhibited higher bacterial numbers at 24 h, a pronounced decline in viability was observed between 24 h and 48 h. In contrast, bacteria encapsulated within alginate beads maintained substantially higher viability at 48 h and continued to exhibit slow growth over this period (Figure 4). These results indicate that the bead environment provides a protective niche that enhances long-term bacterial survival.

### 2.6. Growth of Bifidobacterium Under Aerobic Incubation Conditions

**Figure 5:**
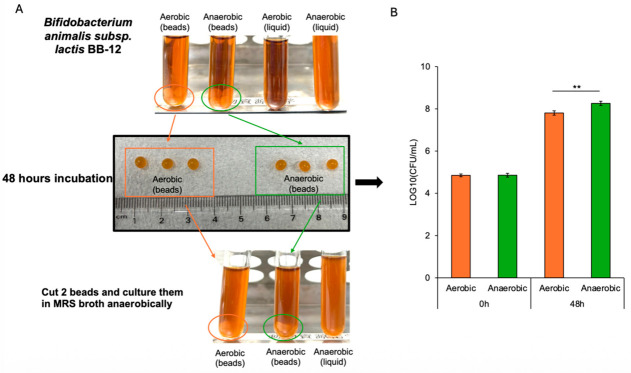
Alginate beads support growth of anaerobic bacteria under aerobic conditions. (**A**) Growth of *Bifidobacterium animalis* subsp. *lactis* BB-12 in liquid MRS medium and alginate beads under aerobic conditions. (**B**) Comparison of bacterial growth in alginate beads under aerobic and anaerobic conditions. The experiment was independently repeated three times (biological replicates). Data are presented as mean ± standard deviation (SD). Statistical analysis was performed using Student’s *t*-test. Differences were considered statistically significant at *p* < 0.05. Data are presented as mean ± SD (n = 3 biological replicates). Statistical significance was determined by Student’s *t*-test. ** *p* < 0.01.

*Bifidobacterium animalis* subsp. *lactis*, a strictly anaerobic bacterium, was encapsulated in alginate beads and incubated under aerobic conditions. Under conventional liquid culture conditions, this strain exhibits limited or no growth in the presence of oxygen. In contrast, bacterial growth was observed when cells were encapsulated within alginate beads, whereas growth remained limited in non-encapsulated controls under the same aerobic conditions (Figure 5A,B). These results suggest that the calcium alginate beads may provide locally protective conditions that support bacterial proliferation despite external oxygen exposure. However, the specific mechanisms underlying this protection were not directly investigated in the present study. Consistent with this interpretation, both *L. paracasei* and *B. animalis* subsp. *lactis* encapsulated within alginate beads exhibited improved survival following exposure to exogenous hydrogen peroxide compared with non-encapsulated controls, indicating that the bead matrix can mitigate oxidative stress.

These findings suggest that calcium alginate beads may provide locally protective microenvironments that support the growth of anaerobic bacteria under otherwise unfavorable conditions.

### 2.7. Alginate Bead Encapsulation Enhances Resistance to Oxidative Stress

**Figure 6:**
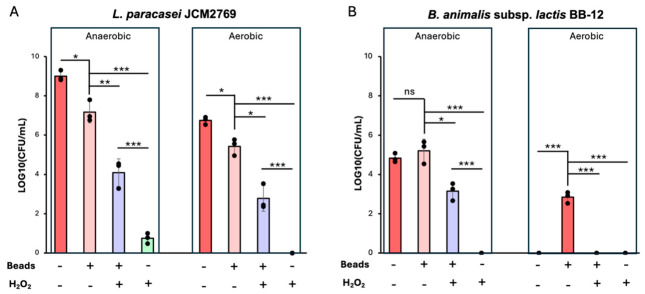
Effect of alginate bead encapsulation on bacterial survival under hydrogen peroxide-induced oxidative stress. (**A**) Viable cell counts of *L. paracasei* JCM2769 and (**B**) *B. animalis* subsp. *lactis* BB-12 cultured under anaerobic or aerobic conditions in the presence or absence of calcium alginate bead encapsulation and hydrogen peroxide treatment. Hydrogen peroxide was added at a final concentration of 0.15‰ (*w*/*v*). Encapsulated bacteria were recovered by citrate-mediated dissolution of alginate beads prior to CFU enumeration. Data are presented as mean ± SD (n = 3 biological replicates). Statistical significance was determined by one-way ANOVA followed by Tukey’s multiple comparison test. ns, not significant; * *p* < 0.05, ** *p* < 0.01, *** *p* < 0.001.

To further investigate whether alginate bead encapsulation protects bacteria from oxidative stress, both *L. paracasei* JCM2769 and *B. animalis* subsp. *lactis* BB-12 were exposed to hydrogen peroxide and viable bacteria were quantified by CFU enumeration after 18 h incubation (Figure 6).

For *L. paracasei*, exposure to hydrogen peroxide significantly reduced bacterial survival under both anaerobic and aerobic conditions. However, encapsulated bacteria maintained substantially higher viable counts than non-encapsulated controls. Similar protective effects were observed for *B. animalis* subsp. *lactis* BB-12. In the presence of hydrogen peroxide, viable bacteria were recovered only from encapsulated cultures, whereas free-cell cultures showed little or no detectable growth. These findings indicate that alginate bead encapsulation markedly improves bacterial tolerance to oxidative stress.

### 2.8. Comparison with Previous Alginate-Based Probiotic Encapsulation Studies

Previous studies have mainly optimized alginate-based encapsulation to protect probiotics during gastrointestinal exposure, storage, freeze-drying, or incorporation into food matrices [11,12]. By contrast, the present study examined alginate beads as a cultivation platform and quantified how the bead matrix influences bacterial localization, release, and post-cultivation recovery. The observation that less than 1% of total *L. paracasei* cells were released during the first 12 h suggests that 2.0% calcium alginate beads provide a strongly confining but metabolically permeable environment. This is distinct from conventional probiotic encapsulation studies, where release is usually evaluated after simulated digestion or during product storage rather than during active growth [28,29,30].

A key distinction between the present study and conventional probiotic encapsulation research is the experimental focus on bacterial behavior during active cultivation rather than post-cultivation protection. Most previous studies have evaluated alginate encapsulation primarily as a strategy to improve storage stability, freeze-drying survival, or resistance to gastrointestinal stress [7]. In contrast, our results demonstrate that calcium alginate beads can function as cultivation microenvironments that spatially regulate bacterial growth, limit early bacterial release, and improve long-term viability while allowing efficient recovery of viable cells. These properties may be advantageous in applications requiring controlled microbial cultivation, including probiotic production, starter culture preparation, immobilized-cell fermentation, and microbial bioprocessing. The ability to maintain bacterial populations within a confined yet metabolically accessible environment distinguishes this system from many conventional encapsulation approaches that primarily serve as passive protective carriers. Rather than functioning solely as protective carriers, calcium alginate beads may serve as controllable cultivation microenvironments that regulate bacterial localization, release dynamics, and long-term viability. These properties highlight their potential utility in microbial cultivation, immobilized fermentation, and probiotic manufacturing applications.

An additional practical advantage of this system is the efficient recovery of viable bacteria through citrate-mediated or alginate lyase-mediated bead dissolution [31]. Rather than serving solely as a terminal delivery vehicle, the alginate bead platform allows bacterial cultivation, retention, and subsequent recovery in a controllable manner. This feature may be particularly valuable for applications requiring downstream use of viable microorganisms, including starter culture production, probiotic manufacturing, immobilized-cell fermentation systems, and the cultivation of oxygen-sensitive microorganisms that can subsequently be released for food, industrial, or research applications. Although the potential physiological effects of citrate exposure on bacterial cells were not investigated in the present study, the recovered bacteria remained viable and cultivable under the conditions tested.

### 2.9. Limitations of the Present Study

This study has several limitations. First, although enhanced growth of encapsulated *B. animalis* subsp. *lactis* under aerobic conditions and increased resistance to hydrogen peroxide suggest that alginate beads provide protection against oxidative stress, oxygen concentrations and gradients within the beads were not directly measured. Therefore, the formation of localized low-oxygen regions remains a hypothesis that requires validation using oxygen-sensitive probes or microsensor-based approaches. Second, the present study focused primarily on the biological effects of alginate encapsulation on bacterial growth, release behavior, and viability. Mechanical properties of the beads, such as Young’s modulus, compression resistance, and stiffness, were not directly quantified, although previous studies have reported improved mechanical stability at higher alginate concentrations. Third, only two bacterial strains were evaluated in this study. Whether similar effects occur in other probiotic, anaerobic, or industrially relevant microorganisms remains to be determined. Finally, all experiments were conducted at laboratory scale under static culture conditions. Future studies should evaluate the performance of this system under larger-scale cultivation settings and investigate the influence of flow conditions, oxygen diffusion, metabolite gradients, and hydrogel composition on bacterial behavior.

### 2.10. Future Perspectives and Potential Application

From a practical perspective, the encapsulation system described here may also be suitable for larger-scale applications. Calcium alginate beads can be fabricated using inexpensive food-grade materials and established industrial encapsulation technologies, including extrusion-based and nozzle-assisted bead generation systems. The ability of the beads to retain bacteria while permitting nutrient diffusion, together with the efficient recovery of viable cells through citrate-mediated dissolution, may facilitate applications in probiotic production, starter culture preparation, immobilized fermentation, and other microbial cultivation processes. Nevertheless, additional studies will be required to evaluate system performance under large-scale manufacturing conditions and in continuous cultivation settings.

The modular nature of the alginate bead platform may also facilitate the development of co-culture and multispecies microbial systems. In principle, different microorganisms could be encapsulated separately or in combination to create spatially organized microbial consortia while maintaining controllable cultivation and recovery. Such approaches may be relevant for probiotic formulations, starter cultures, and engineered microbial communities. However, the feasibility and biological consequences of multispecies encapsulation were not investigated in the present study and warrant future research.

## 3. Conclusions

In this study, we evaluated calcium alginate beads as a physical cultivation environment for *L. paracasei* and investigated their effects on bacterial growth behavior, release dynamics, and viability. Our results demonstrate that alginate concentration plays a critical role in determining bead properties. Increasing alginate concentration to 2.0% improved structural stability and reduced permeability, which is consistent with previous reports showing that higher alginate concentrations generate denser hydrogel networks with reduced pore size and restricted molecular diffusion [32,33]. The release analysis revealed remarkably slow bacterial escape from the beads during early cultivation, with less than 1% of total bacteria detected in the surrounding medium within the first 12 h. Importantly, release kinetics were determined using independent samples for each time point rather than repeated sampling from the same culture vessel. Therefore, the observed release profiles were not influenced by progressive reductions in culture volume or nutrient availability caused by serial sampling.

Previous immobilized-cell studies have reported gradual release of lactic acid bacteria from alginate matrices during fermentation [34]; however, many of these studies primarily evaluated release from the perspective of production stability or downstream fermentation performance. In contrast, the present study quantitatively compared bacterial populations inside and outside the beads over time, allowing evaluation of bacterial confinement during active proliferation. The extremely low early release observed here suggests that 2.0% calcium alginate beads create a highly restrictive physical environment while still permitting nutrient and metabolite diffusion. This combination of metabolic permeability and cellular confinement may contribute to the prolonged maintenance of bacterial viability observed in this study.

Although this conclusion is based on indirect observations, the combination of bead turbidity and relatively clear surrounding medium suggests that the bead structure influences the localization of bacterial proliferation. The release analysis indicated that only a small fraction of bacteria was detected in the surrounding medium during early cultivation, followed by a gradual increase over time. Rather than defining this behavior using categorical terms, our results support a model in which alginate beads modulate bacterial release in a time-dependent manner. This behavior is likely influenced by the balance between cell proliferation within the beads and diffusion through the hydrogel matrix. Encapsulation was also associated with improved maintenance of viable cell counts compared with conventional liquid culture. One possible explanation is that the bead structure provides a buffered microenvironment that mitigates rapid environmental changes [35], such as nutrient depletion or metabolite accumulation. However, further studies are required to clarify the underlying mechanisms.

However, unlike conventional probiotic encapsulation studies that mainly evaluate post-storage or post-digestion survival, the present study investigated bacterial viability during active growth phases. The slower decline in viable counts observed within alginate beads suggests that the hydrogel matrix may buffer environmental fluctuations associated with conventional liquid culture, including rapid nutrient depletion, metabolite accumulation, or local pH stress.

An interesting observation was that *B. animalis* subsp. *lactis* exhibited detectable growth under aerobic incubation when encapsulated within alginate beads. Previous studies have reported that hydrogel encapsulation can partially protect oxygen-sensitive probiotics during storage or gastrointestinal transit by limiting environmental oxygen exposure [36,37]. Our observations suggest that alginate encapsulation creates a protective microenvironment that enhances bacterial tolerance to aerobic conditions. Additional experiments performed during manuscript revision demonstrated that encapsulated *L. paracasei* and *B. animalis* subsp. *lactis* exhibited improved resistance to exogenously added hydrogen peroxide compared with non-encapsulated controls. These findings indicate that the bead matrix can attenuate oxidative stress experienced by encapsulated bacteria. Nevertheless, oxygen concentrations within the beads were not directly measured. Therefore, we cannot conclude that low-oxygen niches were formed. Instead, we propose that reduced oxygen exposure, restricted diffusion of reactive oxygen species, or other physicochemical properties of the hydrogel matrix may contribute to the observed protective effect.

In conclusion, our results suggest that calcium alginate beads can function as a controllable physical environment that influences bacterial growth, release, and viability under the tested conditions. This system may provide a useful platform for studying microbial behavior in confined environments and could have potential applications in controlled fermentation and related fields.

## 4. Materials and Methods

### 4.1. Bacterial Strains and Culture Conditions

*Lacticaseibacillus paracasei* JCM 2769 (Japan Collection of Microorganisms, RIKEN, 3-1-1 Koyadai, Tsukuba, Ibaraki 305-0074, Japan) was used as the primary model strain. *Bifidobacterium animalis* subsp. *lactis* BB-12 (Fuji Photo Film Co., 7-3 Akasaka 9-chome, Minato-ku, Tokyo 107-0052, Japan) was used to evaluate bacterial growth under aerobic incubation conditions.

Bacteria were cultured in de Man, Rogosa and Sharpe (MRS) medium (BD Biosciences, Franklin Lakes, NJ 07417-1880, USA) at 37 °C under static conditions. For anaerobic cultivation, an anaerobic chamber (or anaerobic jar, if applicable) was used when required. Colony-forming units (CFUs) were determined after incubation for 48 h on MRS agar plates.

### 4.2. Preparation and Sterilization of Sodium Alginate Solutions

Sodium alginate (food grade; Fujifilm Wako, Osaka 541-0045, Japan) was dissolved in sterile deionized water at concentrations of 1.0%, 1.5%, or 2.0% (*w*/*v*). Due to the high viscosity of alginate solutions and the potential degradation of polymer chains during autoclaving, thermal sterilization was not applied. Sodium alginate solutions were prepared in sterile containers and exposed overnight to the built-in UV lamp of a biological safety cabinet under aseptic conditions. Following UV treatment, blank alginate beads were prepared without bacterial inoculation and incubated in sterile MRS medium. After incubation, the beads were dissolved using sodium citrate and plated on MRS agar for contamination assessment. The effectiveness of UV sterilization was further evaluated experimentally. Sterilized alginate solutions without bacterial inoculation were processed into calcium alginate beads and incubated in sterile MRS medium under the same conditions used for bacterial cultivation. After incubation, the beads were dissolved using sodium citrate and the resulting suspensions were plated on MRS agar. No bacterial colonies were detected after 48 h incubation, indicating the absence of detectable microbial contamination under the conditions tested (Figure S2). For visualization purposes, Congo Red (Lot#43021, Muto Pure Chemicals Co., Ltd., Tokyo 113-0033, Japan) was added to alginate solutions at a final concentration of 1% (*w*/*v*). Congo Red was used solely as a tracer and was not involved in any functional assays.

### 4.3. Fabrication of Calcium Alginate Beads

Calcium alginate beads were prepared by extruding sodium alginate solution dropwise through a sterile 1 mL syringe into a 1.5% (*w*/*v*) calcium chloride (CaCl_2_) solution. Upon contact, droplets gelled immediately to form spherical beads. Beads were allowed to crosslink for 30 min at room temperature, then collected and washed three times with sterile phosphate-buffered saline (PBS) to remove excess calcium ions. The number of beads generated per milliliter of alginate solution was recorded to estimate the approximate volume of alginate per bead.

To characterize bead size, the diameters of calcium alginate beads were measured immediately after preparation and after cultivation. Individual bead diameters were determined using a digital caliper (BLD-100, Niigata Seiki Co., Ltd., Niigata 950-2253, Japan). For each condition, multiple beads were randomly selected and measured individually.

### 4.4. Permeability Assessment of Alginate Hydrogels

Alginate hydrogels of different concentrations were coated onto membrane filters to form uniform layers. A 1% (*w*/*v*) crystal violet solution was added to the upper chamber, and after incubation, the permeated solution was collected from the lower chamber. The absorbance of the permeated solution was measured at 560 nm using a microplate reader (PerkinElmer, Waltham, MA 02451, USA). Filters without alginate coating were used as controls (100% permeability). Relative permeability was calculated as the percentage of absorbance compared to control conditions.

### 4.5. pH Stability of Calcium Alginate Beads

Beads prepared from different alginate concentrations were incubated in solutions adjusted to various pH values using HCl or NaOH. After 1.5 h at room temperature, bead morphology was visually assessed. Stability was defined as maintenance of spherical structure without visible fragmentation or dissolution.

### 4.6. Encapsulation and Cultivation of Bacteria

*L. paracasei* cultures were grown to an optical density at 600 nm (OD_600_) of approximately 1.0. Cells were harvested by centrifugation (1 mL culture) and resuspended in 50 µL sterile PBS. The concentrated bacterial suspension was mixed with 10 mL alginate solution to form a homogeneous mixture. The mixture was used to generate bacteria-containing alginate beads as described above. Encapsulated bacteria were cultured in MRS medium at 37 °C under static conditions.

### 4.7. Citrate-Mediated Dissolution of Alginate Beads

To recover encapsulated bacteria, beads were incubated in sodium citrate solution (1.62 g per 100 mL, without pH adjustment) at room temperature for approximately 2 h until complete dissolution was observed. Following dissolution, bacterial cells were collected and immediately subjected to CFU enumeration on MRS agar plates.

### 4.8. Quantification of Bacterial Release Dynamics

To assess bacterial release, a single alginate bead (~50 µL) was incubated in 1 mL MRS medium at 37 °C. At designated time points (2, 4, 6, 8, 10, 12, 24, and 48 h), 50 µL of the surrounding medium was removed for CFU determination.

Independent samples were prepared for each time point. After collection of the supernatant and bead for CFU determination, the corresponding sample was discarded and was not used for subsequent measurements. Therefore, no repeated sampling was performed on the same culture system. After sampling, the remaining bead was dissolved using sodium citrate as described above to quantify the bacteria retained within the bead. CFUs were determined by serial dilution and plating. The release rate was calculated as the proportion of CFUs detected in the surrounding medium relative to the total CFUs (medium + bead).

For bead disruption, calcium alginate beads were aseptically cut using a sterile 18-gauge (18G) needle. The cut beads were immediately transferred to fresh sterile tubes using the same sterile needle to avoid external contamination. Intact beads, mechanically disrupted beads, and corresponding culture supernatants were then incubated in MRS medium under identical conditions.

### 4.9. DAPI Staining of Alginate Beads

To visualize bacterial localization within alginate beads, sterile and bacteria-containing alginate beads were incubated with 4′,6-diamidino-2-phenylindole (DAPI) solution (final concentration = 1 μg/mL) prepared by diluting the stock reagent in sterile distilled water according to the manufacturer’s instructions. Beads were stained for 30 min at room temperature in the dark and subsequently washed with sterile distilled water to remove excess dye. Fluorescence images were acquired using a fluorescence microscope (KEYENCE BZ-X800, Keyence Corporation, Osaka 533-8555, Japan) under identical imaging conditions.

### 4.10. Scanning Electron Microscopy (SEM)

Beads were fixed in 2% (*v*/*v*) glutaraldehyde overnight. Samples were dehydrated sequentially in 50%, 70%, 90%, and 99% ethanol for 20 min each, followed by immersion in t-butyl alcohol. Samples were coated with platinum–palladium and observed using a scanning electron microscope (HITACHI SU8000, Hitachi High-Technologies Corporation, Tokyo 105-6409, Japan).

### 4.11. Comparison of Bacterial Viability

Bacterial viability was compared between encapsulated and free liquid culture conditions. For encapsulated conditions, one alginate bead (~50 µL) was used as the experimental unit. For liquid cultures, 50 µL culture volume was used as an equivalent reference. After 24 and 48 h of incubation, CFUs were determined. Experiments were performed using independent biological replicates.

### 4.12. Evaluation of Oxidative Stress Tolerance

To evaluate the protective effect of alginate bead encapsulation against oxidative stress, hydrogen peroxide (H_2_O_2_) challenge experiments were performed using both *L. paracasei* JCM2769 and *B. animalis* subsp. *lactis* BB-12. A 6% (*w*/*v*) H_2_O_2_ stock solution (MCO-H2O2-PJ, Yamato Co., Ltd., Tokyo 104-6136, Japan) was diluted in MRS medium to achieve the desired final concentration. For each condition, 500 μL of bacteria-containing alginate beads or an equivalent volume of free-cell culture was added to 5 mL of MRS medium containing H_2_O_2_. Cultures were incubated for 18 h under either aerobic or anaerobic conditions. Based on the screening experiment shown in Figure S3, a final H_2_O_2_ concentration of 0.15‰ (*w*/*v*) was selected for quantitative analysis.

Following incubation, culture supernatants were collected. Alginate beads were then dissolved by incubation in 5 mL sodium citrate solution, and the resulting bacterial suspension was combined with the previously collected culture medium. Serial ten-fold dilutions were prepared in PBS, and viable bacteria were quantified by CFU enumeration on MRS agar plates. All experiments were performed using three independent biological replicates (n = 3).

### 4.13. Statistical Analysis

All experiments were independently repeated three times (n = 3 biological replicates). Data are presented as mean ± standard deviation (SD). Technical replicates were not included. Data are presented as mean ± standard deviation (SD). Because of the limited sample size, formal normality testing was not performed. Statistical comparisons were conducted using one-way ANOVA followed by Tukey’s multiple comparison test or Student’s *t*-test, as indicated in the figure legends.

## Data Availability

All data generated or analyzed during this study are included in this published article and its Supplementary Materials. Raw datasets generated during the current study are available from the corresponding author upon reasonable request. No large-scale sequencing datasets or external repository submissions were generated in this study.

## Supplementary Materials

The following supporting information can be downloaded at: https://www.mdpi.com/article/10.3390/gels12060518/s1, Figure S1. Diameter changes of 2.0% calcium alginate beads during cultivation. The diameters of individual calcium alginate beads were measured after bead formation (0 h) and after 12 h of anaerobic incubation in MRS medium at 37 °C. Each colored dot represents an individual bead, and paired lines connect measurements from the same bead. Bars represent the mean diameter of all measured beads (n = 8). Statistical significance was determined using a paired Student’s *t*-test. *** *p* < 0.001; Figure S2. Sterility validation of UV-treated alginate preparations. (A) Sterility assessment of sodium alginate solutions following overnight UV irradiation. UV-treated alginate solution was plated onto MRS and LB agar plates and incubated aerobically at 37 °C overnight. No bacterial growth was observed on either medium. (B) Verification that UV-treated alginate remained suitable for bacterial cultivation. Following overnight UV irradiation, *L. paracasei* JCM2769 was inoculated into the alginate solution and plated onto MRS agar. Robust bacterial growth was observed in the inoculated control, whereas no growth was detected in the non-inoculated UV-treated alginate control. These results support the effectiveness of the UV sterilization procedure under the conditions used in this study; Figure S3. Effect of hydrogen peroxide on the growth of free and alginate bead-encapsulated probiotic bacteria. (A) Growth of *L. paracasei* JCM2769 and (B) *B. animalis* subsp. lactis BB-12 cultured in calcium alginate beads under anaerobic and aerobic conditions in the presence of increasing concentrations of hydrogen peroxide. Free-cell cultures were included as controls under identical conditions. Hydrogen peroxide concentrations shown in panel (C) represent final concentrations in the culture medium (0, 0.15, 0.30, 0.60, 1.20, and 2.40‰, *w*/*v*). Representative images were acquired after incubation under the indicated cultivation conditions.
