# Supplementary material for: Encapsulation in Calcium Alginate Beads Regulates Growth, Release and Viability of Probiotic Bacteria Through Protective Microenvironments
Source: Gels. 2026 Jun 10;12(6):518. doi: 10.3390/gels12060518 (PMC13298036; doi:10.3390/gels12060518)
Supplement: Supplementary file 1 [file gels-12-00518-s001.zip › gels-4352540-supplementary.pdf]

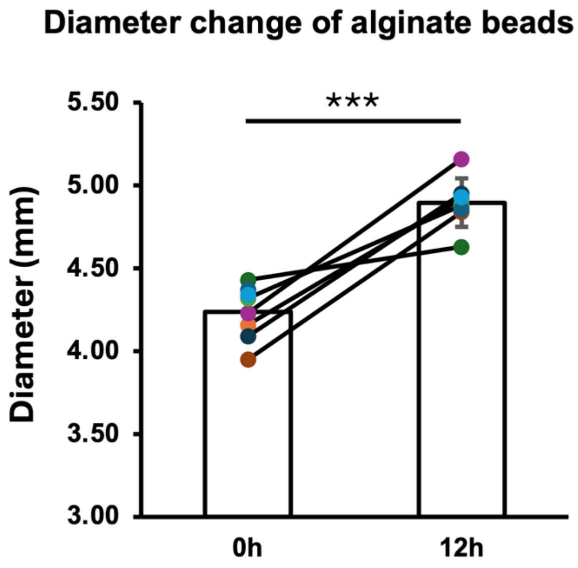

Figure S1. Diameter changes of 2.0% calcium alginate beads during cultivation.

The diameters of individual calcium alginate beads were measured after bead formation (0 h) and after 12 h of anaerobic incubation in MRS medium at 37 °C. Each colored dot represents an individual bead, and paired lines connect measurements from the same bead. Bars represent the mean diameter of all measured beads (n=8). Statistical significance was determined using a paired Student's *t-test*. *** $p < 0.001$.

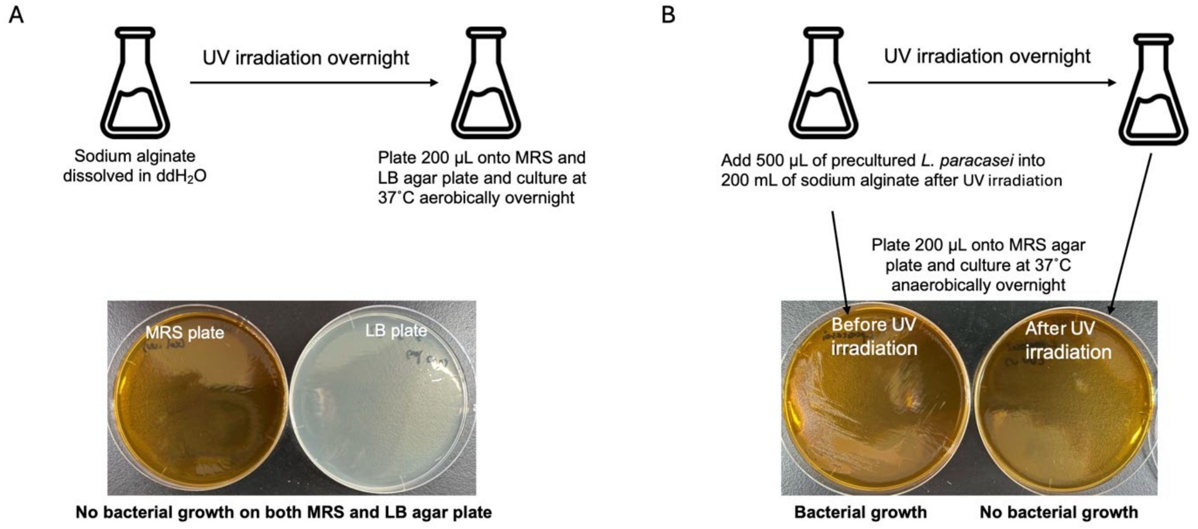

Figure S2. Sterility validation of UV-treated alginate preparations.

(A) Sterility assessment of sodium alginate solutions following overnight UV irradiation. UV-treated alginate solution was plated onto MRS and LB agar plates and incubated aerobically at 37 °C overnight. No bacterial growth was observed on either medium. (B) Verification that UV-treated alginate remained suitable for bacterial cultivation. Following overnight UV irradiation, *L. paracasei* JCM2769 was inoculated into the alginate solution and plated onto MRS agar. Robust bacterial growth was observed in the inoculated control, whereas no growth was detected in the non-inoculated UV-treated alginate control. These results support the effectiveness of the UV sterilization procedure under the conditions used in this study.

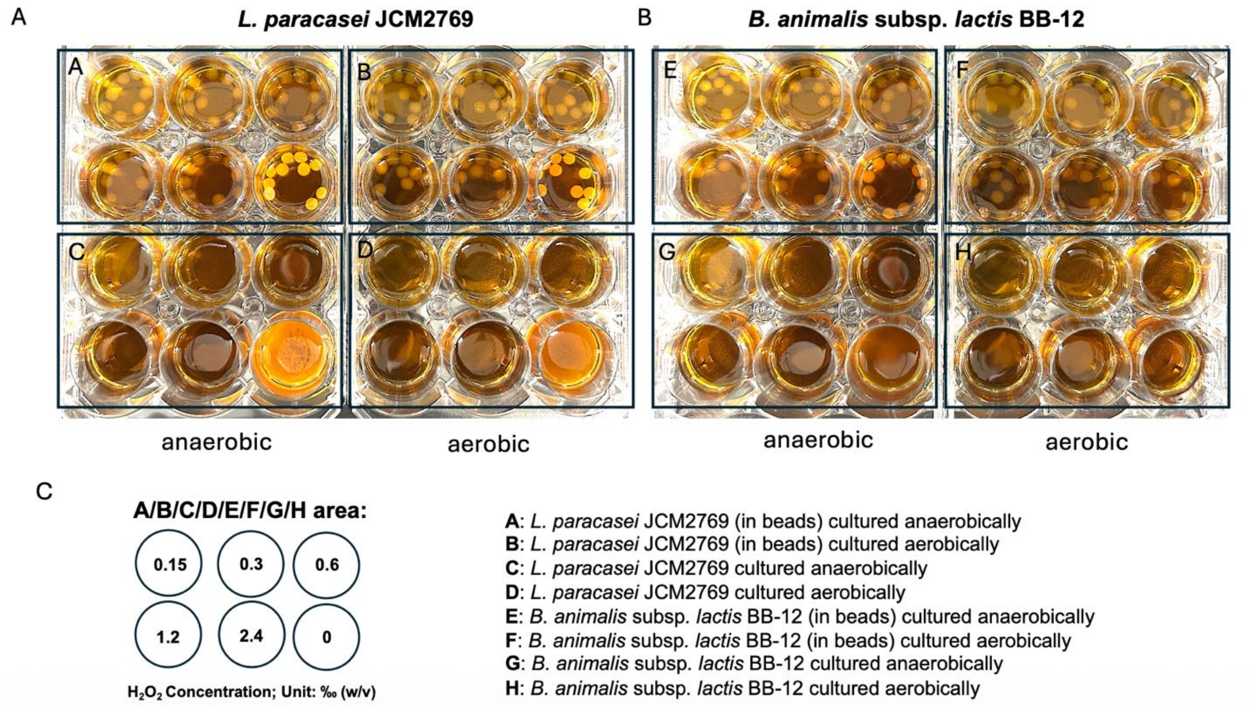

Figure S3. Effect of hydrogen peroxide on the growth of free and alginate bead-encapsulated probiotic bacteria.

(A) Growth of *L. paracasei* JCM2769 and (B) *B. animalis* subsp. *lactis* BB-12 cultured in calcium alginate beads under anaerobic and aerobic conditions in the presence of increasing concentrations of hydrogen peroxide. Free-cell cultures were included as controls under identical conditions. Hydrogen peroxide concentrations shown in panel (C) represent final concentrations in the culture medium (0, 0.15, 0.30, 0.60, 1.20, and 2.40‰, w/v). Representative images were acquired after incubation under the indicated cultivation conditions.
